# Elevated Serum Pancreatic Enzymes in a Patient With Acute Epigastric Pain and End-Stage Renal Disease

**DOI:** 10.7759/cureus.24315

**Published:** 2022-04-20

**Authors:** Ghania Masri, Alya Barq

**Affiliations:** 1 Department of Medicine, University of Florida College of Medicine – Jacksonville, Jacksonville, USA; 2 Department of Medicine, University of Florida College of Medicine, Gainesville, USA

**Keywords:** nausea and vomiting, end stage renal disease, cyclic vomiting syndrome, lipase, acute pancreatitis

## Abstract

Acute pancreatitis is one of the most common diagnoses for patients admitted to the hospital with acute abdominal pain and nausea and vomiting. often elevated amylase and lipase support the diagnosis. This case illustrates the importance of recognizing the elevated laboratory findings seen in patients with end-stage renal disease, especially those laboratory findings that aid in making clinical decisions and/or establishing the diagnoses. We present a case of a patient misdiagnosed with recurrent acute pancreatitis due to his recurrent episodes of nausea, vomiting, abdominal pain, and persistently elevated pancreatic enzymes in the setting of end-stage renal disease. It is important for clinicians to recognize that these enzymes are renally eliminated and thus will be elevated as a result of the renal disease, which would limit the use of pancreatic enzymes to establish the diagnosis of acute pancreatitis.

## Introduction

Acute pancreatitis is one of the most common reasons for inpatient admission secondary to gastroenterology disease, and accurate diagnosis requires two of three criteria, including acute abdominal pain, elevated serum lipase and amylase levels equal to or higher than three times the upper limit of normal and abdominal CT criteria demonstrating acute pancreatitis [1]. Here we describe the case of a 30-year-old male with end-stage renal disease on peritoneal dialysis and a presumptive history of recurrent pancreatitis presented to the emergency department with acute severe epigastric pain associated with nausea and frequent bouts of vomiting. Initial laboratory workup was significant for a lipase of 210 U/L, given these findings, suspicion for an episode of acute pancreatitis was high. Computed tomography of the abdomen and pelvis with IV contrast showed a normal pancreas with no focal abnormality. These findings were inconsistent with the diagnosis and disease course of acute pancreatitis given the fact that elevated pancreatic enzymes are common in end-stage renal disease in the presence of acute pancreatitis.

## Case presentation

A 30-year-old male with PMH of end-stage renal disease secondary to type 1 diabetes mellitus on peritoneal dialysis, hypertension, and a history of recurrent pancreatitis presented to the emergency department with acute severe epigastric pain associated with nausea and frequent bouts of vomiting that began the morning of the presentation. He described the vomitus as green and denied any red or coffee-ground emesis. He denied melena, hematochezia, fever, chills, chest pain, or SOB. He endorsed previous hospitalizations for pancreatitis at outside hospitals, the last one being a year ago. He stated that he does peritoneal dialysis every other day, but he did not undergo dialysis since the week prior to presentation due to his catheter malfunctioning. He endorsed daily cannabis use but denied alcohol or other illicit/recreational drug use.

On physical exam, his vital signs were significant for elevated blood pressure 233/140, and the abdomen was soft, with tenderness to palpation in the epigastrium. His peritoneal dialysis catheter was in place and the site was clean, dry, and intact. There were no signs concerning peritonitis. He was given a 1-liter bolus of lactated ringers and Zofran for nausea. In the emergency department, labs were significant for a lipase of 210 U/L reference range (0-60 U/L), creatinine of 4 mg/dL reference range (0.67-1.17 mg/dL), and BUN of 56 mg/dL reference range (7-25 mg/dL). Given his subjective history of pancreatitis at outside hospitals, elevated lipase, and severe epigastric pain associated with nausea and vomiting, initial suspicion for an episode of acute pancreatitis was high and he was admitted to the internal medicine service for further management. The day after admission he rapidly improved, with a resolution of pain, nausea, and improvement of appetite. His serum lipase level had decreased to 84 U/L after peritoneal dialysis. CT abd/pelvis showed normal pancreas with no focal abnormality and peritoneal dialysis catheter was identified within the left lower quadrant. These findings were inconsistent with the diagnosis and disease course of acute pancreatitis [1], thus our clinical suspicion of that diagnosis decreased. We then considered alternative diagnoses including cyclic vomiting syndrome secondary to cannabis use, and cannabinoid hyperemesis syndrome.

## Discussion

The diagnosis of acute pancreatitis requires the presence of two out of three criteria including elevation of pancreatic enzymes three times the upper limit of normal, radiologic findings suggestive of the diagnosis, and the classic clinical presentation [1]. In patients with end-stage renal disease, serum levels of pancreatic enzymes may be elevated in the absence of acute pancreatitis. Glomerular filtration is the main mechanism for the removal of lipase from the serum, thus explaining the elevated levels seen in patients with renal disease [2-4]. In patients with end-stage renal disease presenting with signs and symptoms concerning acute pancreatitis, this must be taken into consideration. The patient described in this case has had recurrent bouts of epigastric abdominal pain and it appears he had been misdiagnosed with acute pancreatitis at each hospitalization likely due to his persistently elevated pancreatic enzymes secondary to advanced renal disease.

CT with IV contrast is a very reliable imaging modality to diagnose the severity of acute pancreatitis and to identify complications of pancreatitis. Patients with acute pancreatitis usually have enlargement of the pancreas with diffuse edema and heterogeneity of pancreatic parenchyma [1]. In this case, the patient’s contrast computed tomography revealed a decisively normal pancreases appearance with an unremarkable gallbladder without intrahepatic or extrahepatic biliary dilation.

Given the fact that the patients' symptoms improved within 24 hours of admission and unremarkable findings on his contrast abdominal CT despite his presumed history of multiple acute pancreatitis in the past, another more likely diagnosis such as cyclic vomiting syndrome vs cannabinoid hyperemesis syndrome was overlooked [5,6].

This case illustrates the importance of recognizing the altered laboratory findings seen in patients with end-stage renal disease, especially those laboratory findings that aid in making clinical decisions and/or establishing diagnoses. The patient described in this case had been falsely diagnosed with recurrent acute pancreatitis due to his recurrent episodes of nausea, vomiting, abdominal pain, and persistently elevated pancreatic enzymes in the setting of end-stage renal disease.

## Conclusions

Several laboratory parameters including serum pancreatic enzymes are altered in advanced renal disease. Serum lipase and amylase are primarily renally cleared and will often be elevated at baseline in patients with end-stage renal disease. Results in these cases can be difficult to interpret and may be misleading, thus clinicians must be aware of the effect of end-stage renal disease on pancreatic enzyme levels to avoid making an erroneous diagnosis or missing some entirely.

## References

[1] Banks PA, Freeman ML (2006). Practice guidelines in acute pancreatitis. Am J Gastroenterol.

[2] Hameed AM, Lam VW, Pleass HC (2015). Significant elevations of serum lipase not caused by pancreatitis: a systematic review. HPB (Oxford).

[3] Robitaille R, Lafrance JP, Leblanc M (2006). Altered laboratory findings associated with end-stage renal disease. Semin Dial.

[4] Jiang CF, Ng KW, Tan SW, Wu CS, Chen HC, Liang CT, Chen YH (2002). Serum level of amylase and lipase in various stages of chronic renal insufficiency. Zhonghua Yi Xue Za Zhi (Taipei).

[5] Venkatesan T, Levinthal DJ, Li BU (2019). Role of chronic cannabis use: cyclic vomiting syndrome vs cannabinoid hyperemesis syndrome. Neurogastroenterol Motil.

[6] Kumar N, Bashar Q, Reddy N (2012). Cyclic vomiting syndrome (CVS): is there a difference based on onset of symptoms--pediatric versus adult?. BMC Gastroenterol.

